# American Medical Association, Section on Stomatology

**Published:** 1903-01

**Authors:** 


### AMERICAN MEDICAL ASSOCIATION, SECTION ON STOMATOLOGY.

(Continued from Vol. XXIII., page 927.)

*Final Session, Thursday Afternoon, June 12, 1902.*

The meeting was called to order by the Chairman, Dr. A. H. Peck.

A paper entitled "The Legal Status of the Term 'Reputable' as applied to Dental Colleges" was read by Dr. Charles C. Chittenden, D.D.S., of Madison, Wis.

(For Dr. Chittenden's paper, see Vol. XXIII., page 581.)

DISCUSSION.

*Dr. G. V. I. Brown.*—It is important for us to know that this is the first time that we have been able to take up the discussion of the legislation of the dental curriculum and such changes as we desired to have made for the advancement of the profession with a certainty that we could carry them out. The term "reputable" is now defined to mean a standard which is in accordance with the standard of the best institutions. That, too, might be extended to imply that a proper curriculum is one in accordance with what is laid down by those best versed in the educational needs of dental colleges. It seems to me now like opening a vista before us in the work to be done and which this Section has been interested in for so many years. We now come to the consideration of what branches should be taught, how much and how little; what is best adapted to the need of a dentist from the stand-point of the artisan purely, and in order to rank among the professional men of the world as this Section would have him do, taking his rightful place in the rank of medical practitioners. It seems to me that it is a very beautiful thing to receive this crowning of the labors of Dr. Chittenden and of the other gentlemen who have carried on the work of the Section with high ideals, and to know that these aims are in a fair way to be consummated.

*Dr. M. L. Rhein.*—I feel that all of the profession are indebted to Dr. Chittenden for the work of bringing this matter to such a magnificent consummation in his own State. One of the things we have been striving for in our State is to get a unity of understanding so that the bearer of a license to practise in one State could practise in another. We hope that the present interchange of right to practise may extend to other States. It is an unwarranted hardship to ask a man who has been practising for some years to pass the ordinary examination. However, it would be folly for us to take into some of our States practitioners with the indorsement of some of the boards of examiners.

*Dr. A. E. Baldwin.*—I am glad that Dr. Chittenden has been able to present this paper to us and to the profession at large through the *Journal of the American Medical Association* and the INTERNATIONAL DENTAL JOURNAL, because it is a move in the direction of higher standards, higher education, and broader qualifications. To my mind it marks an epoch which will be more readily recognized in the future. It seems to me that this so-called reciprocity in professional work should obtain. It is in every way reasonable and equitable, and, once established, I believe it will do more for the elevation of dentistry than any other one thing. I feel that I can heartily endorse the tribute of Dr. Brown to Dr. Chittenden. For fear of it being misunderstood by the readers of this paper, I want to say that I do not think this decision of the Supreme Court of Wisconsin was qualified in any way by the personal opinion of the Supreme Court judge for Dr. Chittenden. I do not think that was the meaning of Dr. Rhein. The decision of the court will be even more far-reaching than is perhaps now realized.

*Dr. Rhein.*—I trust that my remarks did not bear the interpretation suggested by Dr. Baldwin. I meant that the record of our specialty coming through such a man as Dr. Chittenden proved that we are in a position to receive such a decision.

I would like to say, in regard to the troubles in Germany last year, and which were taken up by our Consul, that in returning last summer from Germany one of our passengers was Professor Andrew D. White, one of our ambassadors to Germany. It was a matter to me of great delight to see the interest taken in our specialty by Professor White, and, desiring to send a message to those interested in this work in America, he said he wanted us to know that in anything in the nature of international complications he placed himself at our disposal, and wanted us to feel that we would have perfect access to him in such matters as had come up last year.

*Dr. Talbot.*—It seems to me that if the State boards adopt the law as explained by Dr. Chittenden, they have an immense power to wield in regard to dental colleges. The word "reputable," and in another clause "the burden of proof placed upon the graduate as to the reputability of the college," covered the ground very nicely. It relieves the State boards of a great deal of hard work and at the same time gives them the ability to maintain high standards. In taking this position the judges seemed to have insight of the conditions and affairs, and have made it plain that any State board with the least amount of ability could place their board in a position to command the respect of all the colleges in the country.

*Dr. Brown.*—As a matter of fact, reciprocity is a possible, and can easily be an actual, fact. I do not suppose any of you will remember the Chairman's Address which I read at Columbus, but in that I outlined the possibility of an arrangement whereby all individual boards can be guided and supervised by the National body, and I made the statement that though the individual board might be mercenary and ignorant, and sometimes the result of political appointment, the selected board from the best representatives of all of the boards of the United States would be as nearly fair and honorable as would be possible to secure. The board of Wisconsin has made all that possible. Under the direction of their attorney the Supreme Court prepared for these their own rules. Instead of being guided by the National body they have adopted as their rules certain rules and regulations based upon the rules of this Association. Under the decision of that court they can require just exactly whatever the rule is, and as they see fit can raise the standard. The board is a judicial body, and in the exercise of its proper powers cannot be overruled. If the different boards choose to adopt similar rules and regulations, under these rules the standard is at once uniform and it is possible for men to go from one State to another without being harassed by examinations. On the other hand, it takes away the possibility of having a too haphazard method.

A part of our work for the future should be to prepare a way for the proper education of the dental student, for a wider medical education, and for his full recognition as a reputable dental practitioner.

*Dr. Williams.*—If we can get good men on the State examining boards there will be no difficulty, but in some States the boards are ruled by politics. I think it is quite proper that the examining boards should have the legal power if they are well qualified and honest men.

*Dr. A. H. Peck.*—The subject has been so entirely covered that I will simply say this, because I am anxious to go on record before the dental world and before the medical world as one who is in favor of everything which tends to uplift our profession. This has been my sentiment for years, and I am exceedingly glad that we have one man who has been on a State board of dental examiners for years, who has given the best part of his energy to the consummation of some plan as a standard for the future. Dr. Chittenden is the man, and he has given us to-day a careful *résumé* of the laws of the various States and their attitude towards dentistry.

It is for us to say whether a school is reputable or not. I hope, further, that the power may be placed with them of determining the value or the reputability of a diploma given to a graduate by any institution. Men and women are turned out of our institutions with their diplomas who are not capable or should not come before the world as graduates of any reputable institution. So long as I hold a guiding hand in the affairs of an institution my efforts shall be in this direction,—to uphold every effort of the State boards throughout the country looking to the elevation of the standard of dentistry.

*Dr. Eames.*—I heartily endorse the statements so far as I have heard them, especially the remarks of the chairman, and, so far as I have looked over the paper, there is a great deal to be done with the State boards themselves, in my opinion, for certainly their methods of examination and questions that they have proposed, whenever they could be ascertained, have been far below what I should term a reputable standard.

Any further word that I might say would simply, if possible, make emphatic the suggestion of the unification of these laws and standards. When the standards of examiners shall be in unison, then it seems to me it would be a very easy matter to enact a law which shall conform to them.

*Dr. Baldwin.*—If it would be in order, it seems to me it would be proper for a resolution of recommendation to go from this Section to the National Board of Dental Examiners requesting, in the name of the Section on Stomatology of the American Medical Association, that they look with favor upon this interpretation given by the Wisconsin Supreme Court.

*Dr. Chittenden* (closing the discussion).—The difficulty in the past has been that the boards of examiners were antagonized by the schools, and there was a spirit of commercialism in the faculties themselves, and it was only after a serious struggle that it was possible for the examiners to get the colleges themselves to set a definite requirement for admission to their schools. From that point we have gone forward, and the years intervening have brought the two institutions more closely together. Last year the faculties associations recognized that the examining boards were really becoming their friends. The suggestions which I have endeavored to show you are absolutely feasible for the establishment of a national educational standard which may set the pace for the world. It is understood and known that the State law of New York is the best and of the highest standard in the United States. It has seemed to most men interested in dental matters to be a standard sufficiently high to be sought and chosen as a criterion to which all boards should come. The matter must necessarily move slowly. The boards are scattered throughout all the States of the Union, and it is difficult to get them to co-operate. Once the colleges encourage what I have undertaken to describe, the boards will understand that it is a desirable thing. As soon as the educational standard was permanently established in 1899 commercialism ceased. With the standard as it is, no school can matriculate a man unless he can present credentials acceptable to the State Superintendent of Public Instruction.

I can see no question, Mr. Chairman, that the propositions made in the paper are not absolutely feasible, that they may not be put into action, and in that way enable us to set a standard of dental education for the world.

Dr. A. F. Baldwin, of Chicago, offered the following resolution:

*Resolved,* That the Stomatological Section of the American Medical Association heartily endorses the decision of the Wisconsin Supreme Court as to the judicial powers of the State board as to the determination of what constitutes "reputability;" and we heartily recommend this decision to the National Association of Dental Examiners as a basis for establishing professional reciprocity in our grand country.

On motion, the resolution was adopted.

Dr. G. V. I. Brown, of Milwaukee, Wis., read a paper entitled "General Nervous Manifestations in Relation to the Jaws and Teeth."

(For Dr. Brown's paper, see Vol. XXIII., page 571.)

DISCUSSION.

*Dr. A. E. Baldwin,* Chicago.—I have no doubt that we all see cases in which not enough attention is given to obscure functional disturbances. Within the last two months I have had cases in which I have given temporary relief, and in which I hope for considerable permanent relief from following the suggestions of this paper.

*Dr. Williams.*—We have all seen these cases of grinding of the teeth, but I do not know that I have ever heard the matter so carefully analyzed before as to relations and causes. In one case in which the teeth were quite worn by the grinding the patient said he ground his teeth all night. I think Dr. Brown has treated the matter very scientifically.

*Dr. Eugene S. Talbot.*—I am very much pleased with the paper, for the reason that Dr. Brown has had a large experience with these cases. Every dentist of some years' experience has these cases. The question, however, is, to be able to diagnose them. It is a singular fact that we as dentists have a great many of this class of patients, neurotics and degenerates. We have first the patient with the unbalanced nervous system, and on account of this the conditions mentioned by Dr. Brown develop. He mentioned the fact that the individual has periods of stress in development, which is very true, indeed. It is to a certain extent true that our systems change every seven years; some of the tissues change oftener than that. There are periods in the life of the individual from conception to old age which mark every important epoch in our histories. The first period of stress is at four and one-half months of fœtal life. The second is at the eruption of the first set of teeth, and in the life of the child this is the most important period. There is greater difficulty in tiding the child over the period of teething. At this time the alimentary canal is changing. It is while these changes are going on that we have the neurotic or degenerate child. The third stress is at the time of the eruption of the permanent teeth. It comes on at the sixth year and remains until all the teeth have erupted. It is at this period of evolution that the child is going through the stage of puberty. This, then, is another very marked period of the child's life, for at this time we have the deformities of the dentine and irregularities of teeth to look after. It is very important that in the eruption of permanent teeth and in the correction of irregularities we should know something of the nervous system, because every one of these individuals who have deformities of the teeth and jaws are neurotics. The mere fact that they have irregularities of the teeth indicates that. The next period of stress begins at about the twentieth year and lasts three or four years. The next stress is at the forty-fifth year, when the senile change develops. Here many of the conditions spoken of by Dr. Brown commence to develop. We have the peculiar condition which we call pyorrhœa alveolaris. It is at this time that neurasthenia sets in, and it affects these nervous and degenerate people to a greater extent than normal individuals. Our schools for idiots and the insane asylums will show that nearly every case of these neurotics and degenerates are suffering from decay of the teeth. As the individual goes through life, if he be a neurotic or a degenerate, he is more apt to have these peculiar symptoms of the jaws and teeth as he advances in age.

I have some very interesting cases in the same line and some most remarkable cases that I would like to present, but for want of time I will not do so.

*Dr. Rhein.*—I do not know of any subject presented to the Section which in its practical application is more valuable to the practitioner than that which Dr. Brown has treated so ably. What pleases me most is the direct attitude of the essayist to revert at once to the cause of the disturbance in finding his remedy. I have had, as has had every one of experience, some such cases. There is nothing more satisfactory in results than the solution of this problem when the cause has been removed. The knowledge as to the proper occlusion of the teeth is the key-note to the correction. I have always given much attention to determine the amount of force upon different teeth in such cases. This involved a consideration of all the motions of the teeth. I have made it a rule to replace every missing tooth in order to equably distribute the occlusive force, and I have a series of cases showing satisfactory results. Personally I have had little success with the soft plates worn at night. I have observed in operative work that there is a general habit of leaving the occlusal portions of the posterior teeth almost devoid of their cusps. This renders the proper performance of function impossible. Another point is that necessity of teaching dental students the meaning of physiological occlusion of the jaws. It is an unfortunate thing that very few dentists understand the physiological occlusion not only of the teeth as a whole, but of individual teeth.

*Dr. G. A. Eames.*—It seems almost absurd to remind an intelligent dental practitioner that this subject is an important one. I was much interested in it when I heard the paper by Dr. Brown in Philadelphia, and realized the great benefit which must result to the profession at large by having attention called to it. I am in hearty accord with the paper, and have no criticism to make. The lesson to be drawn is, I think, that it is a good thing to correct these troubles, but a far better thing to be on the watch as guardians of the public health, and not to wait until a patient complains of symptoms, and thus prevent such a disagreeable and suffering condition.

The cause of the trouble may not only be in the mouth, but in some remote part of the body, both agents working together. Our duty lies in watchfulness for the slightest symptom and in the correction of the occlusion that, with our foresight, we may prevent such disastrous consequences.

*Dr. Brown* (closing the discussion).—I wish to thank the gentlemen for their very kindly expressions and to express my appreciation of Dr. Eames in remembering my paper some years ago. It has interested me very much to know that the testing of the force of the jaws is being done by any one. I doubt its practical value in dealing with the cases, because patients in their conscious moments find it impossible to place the jaws in the position through which the irritation comes. Further, I do not think a patient asked to do that could give the amount of stress upon the teeth that would be given in a moment of nervous excitement or when the nerve-centres are excited by some dream.

Prophylaxis is, of course, what this all leads to. All we can hope to do with a paper of this kind is to call attention to the necessity of finding the factor in the nervous disturbance. The most interesting part of my work is in being able to lead these children in the direction which I hope will prevent these troubles. When parents have suffered and their attention is called to the treatment, they are quite willing to bring their children.

Autointoxication is a predisposing factor, and often an exciting cause of these conditions. I have an interesting case, and have seen others illustrative of this cause. The case is that of a young lady whose headaches have almost entirely disappeared, except when she meets a young man whose influence is such that when I see her on the street I can see by certain lines in her face whether she is going to have a headache. Though the patient is in perfect health, she has in a short time all the symptoms of autointoxication.

